# Strong Response of Stem Photosynthesis to Defoliation in *Mikania micrantha* Highlights the Contribution of Phenotypic Plasticity to Plant Invasiveness

**DOI:** 10.3389/fpls.2021.638796

**Published:** 2021-05-05

**Authors:** Jin Zheng, Tai-Jie Zhang, Bo-Hui Li, Wei-Jie Liang, Qi-Lei Zhang, Min-Ling Cai, Chang-Lian Peng

**Affiliations:** ^1^Guangdong Provincial Key Laboratory of Biotechnology for Plant Development, Guangzhou Key Laboratory of Subtropical Biodiversity and Biomonitoring, School of Life Sciences, South China Normal University, Guangzhou, China; ^2^Guangdong Provincial Key Laboratory of High Technology for Plant Protection, Institute of Plant Protection, Guangdong Academy of Agricultural Sciences, Guangzhou, China

**Keywords:** chloroplasts, ETR, *Mikania micrantha*, pigments, plasticity, stem photosynthesis

## Abstract

Phenotypic plasticity affords invasive plant species the ability to colonize a wide range of habitats, but physiological plasticity of their stems is seldom recognized. Investigation of the stem plasticity of invasive plant species could lead to a better understanding of their invasiveness. We performed pot experiments involving defoliation treatments and isolated culture experiments to determine whether the invasive species *Mikania micrantha* exhibits greater plasticity in the stems than do three non-invasive species that co-occur in southern China and then explored the mechanism underlying the modification of its stem photosynthesis. Our results showed that the stems of *M. micrantha* exhibited higher plasticity in terms of either net or gross photosynthetic rate in response to the defoliation treatment. These effects were positively related to an increased stem elongation rate. The enhancement of stem photosynthesis in *M. micrantha* resulted from the comprehensive action involving increases in the Chl *a*/*b* ratio, D1 protein and stomatal aperture, changes in chloroplast morphology and a decrease in anthocyanins. Increased plasticity of stem photosynthesis may improve the survival of *M. micrantha* under harsh conditions and allow it to rapidly recover from defoliation injuries. Our results highlight that phenotypic plasticity promotes the invasion success of alien plant invaders.

## Introduction

Alien species invasion has been recognized as one of the most severe global ecological issues and environmental threats (Vitousek et al., 1996; Mack et al., 2000). It has substantial negative effects on community structure and function and has contributed to global loss of biodiversity (Courchamp et al., 2017). Determining the factors that contribute to the spread of plants in areas outside their native ranges is currently an important issue of research in invasion biology.

Phenotypic plasticity allows plants to cope with complex heterogeneous environments and is thus often cited as an important mechanism of plant invasion (Richards et al., 2006; Zenni et al., 2014). Phenotypic plasticity occurs when a single genotype alters its morphological, physiological and life-history traits in response to changing environmental conditions (Pigliucci, 2001). These adaptive changes can improve plant growth and fitness (Davidson et al., 2011), particularly for clonal plants with low levels of genetic variation (Keser et al., 2014; Geng et al., 2016). According to an analysis of 133 invasive plant species, the invasion mechanisms in approximately 50% of them were related to phenotypic plasticity (Ren and Zhang, 2009).

Plastic changes can occur in various organs (e.g., roots, stems, and leaves). Because the roots and leaves function in resource acquisition, the magnitude of phenotypic plasticity in these organs is critical for plant ecological adaptability (Violle et al., 2009). Because stems are not the major organs that capture resources from the environment, few studies have focused on the effects of stem plasticity on the viability of plant species.

Stems are connected to roots and leaves and function in supporting the leaves, flowers and fruits; transporting water, mineral elements and organic nutrients; and even carrying out photosynthesis, storing photosynthates and functioning as vegetative propagules (Pate and Jeschke, 1995; Nivot et al., 2008). Nearly all green parts of plants can conduct photosynthesis, particularly those of young stems or twigs. Although stem photosynthesis is often lower than leaf photosynthesis, with maximum rates of up to 75% of those of leaf photosynthesis (Pfanz et al., 2002; Ávila et al., 2014), it has important functions in maintaining whole-plant carbon balance (Kocurek et al., 2020), especially in the case of defoliation events caused by insect attacks, leaf fungal pathogens, etc. (Pfanz and Aschan, 2001), or under stress conditions when leaf photosynthesis is limited due to stomatal closure (Bossard and Rejmanek, 1992; Cernusak and Cheesman, 2015). Stem photosynthesis can refix 60–90% of the CO_2_ respired from local tissues (Pfanz et al., 2002; Kocurek et al., 2020) and increase the growth of stems by 10–30% (Cernusak and Hutley, 2011; Bloemen et al., 2016). The carbohydrates produced by stem photosynthesis are involved in maintaining hydraulic function (Sevanto et al., 2014; Bloemen et al., 2016), refilling xylem vessels after embolism (Schmitz et al., 2012) and alleviating xylem vulnerability to cavitation (De Baerdemaeker et al., 2017). Furthermore, the O_2_ released from stem photosynthesis is important for preventing low-oxygen limitations of mitochondrial respiration in metabolically active stem tissues (Wittmann and Pfanz, 2014). Stem photosynthesis is also associated with drought tolerance (Cernusak and Cheesman, 2015; Ávila-Lovera and Tezara, 2018) and is involved in maintaining sap flow flux (Gao et al., 2016). Based on the results of these previous studies, it can be predicted that if a species is capable of dynamically adjusting stem photosynthesis, its viability is greatly enhanced.

*Mikania micrantha* Kunth, commonly known as the ‘mile-a-minute’ weed, is a perennial herbaceous creeping vine belonging to the Asteraceae family and is native to Central and South America (Holm et al., 1977). It has caused substantial economic and ecological losses in plantation crops and commercial and secondary forests within its range of introduction, which includes tropical Asia, Pacific islands, Indian Ocean islands, and Florida in the United States (Waterhouse, 2003; Zhang et al., 2004; Manrique et al., 2011; Day et al., 2016). In China, *M. micrantha* was introduced into Hong Kong in the late 1800s and has since spread throughout southern China (Wang et al., 2003). *M. micrantha* has been listed as one of the 10 worst weeds and one of the 100 worst invasive species in the world (Lowe et al., 2001). It grows extremely fast (up to 20 cm in a 24-h period) (Li et al., 2012), can climb to the top of plant canopies, forms dense thickets, outcompetes existing vegetation by blocking sunlight and releasing allelochemicals (Zhang et al., 2004), and ultimately leads to a loss of species diversity. Moreover, it can alter the soil microbial community structures and soil nutrient cycling in invaded areas (Li et al., 2006; Chen et al., 2009).

The stems of *M. micrantha* are photosynthetic (Liu et al., 2020), and once this species becomes established, it can rapidly expand via stems that creep along the ground or over other plants, forming clonal ramets. In this study, we hypothesized that the stems of *M. micrantha* are more plastic than those of the non-invasive species that co-occur in southern China. To test this hypothesis, we perform pot experiments to investigate whether there is a difference in photosynthesis performance and growth in response to defoliation treatment between *M. micrantha* and non-invasive species and then further revealed the mechanism through which stem photosynthesis is modified in *M. micrantha* by evaluating stomatal aperture, chlorophyll content, chlorophyll fluorescence, photosynthesis-related proteins and chloroplast ultrastructure. We also used isolated culture experiments to analyze the regeneration patterns and photosynthesis potential in stem cuttings of both *M. micrantha* and the non-invasive species.

## Materials and Methods

### Plant Materials

Three non-invasive species (*Pharbitis nil* (L.) Choisy, *Paederia scandens* (Lour.) Merr, and *Pueraria lobata* Ohwi) were selected as the objects to be compared with *M. micrantha*. *Pharbitis nil* (Convolvulaceae) is widely cultivated as ornamental plant in tropical and sub-tropical regions in China, and the vines of this species grow up to 3–4 m long. *Paederia scandens*, commonly known as “JiShiTeng,” is a member of the Rubiaceae family. This species is widely distributed in the south of the Yangtze River in China and its vines grow up to 4–5 m long. *Pueraria lobata* is a leguminous vine native to eastern Asia, commonly known as kudzu. This plant has tuberous roots and its stems grow 10 to 30 m in one growing season (Mitich, 2000). The growth form of both invasive and non-invasive species are herbaceous climbing vines that have green photosynthetic stems all the year round in south China. Vigorous stems of the invasive species and non-invasive species were collected from wild populations near the Guangdong Academy of Agricultural Sciences, Guangzhou, China, and used for vegetative propagation.

### Defoliation Tests

We conducted exploratory tests involving artificial defoliation of the invasive and non-invasive species from April to September in 2019. Stems of the invasive and non-invasive species were cut into approximately 15-cm-long segments that each had one node, and all leaves attached to the stems were removed. The cuttings were cultivated in plastic pots (15 cm in diameter) filled with jiffy substrate (Jiffy Products International B.V., Moerdijk, Netherlands), and placed under shade conditions in the first week. The regenerated plantlets were then grown under greenhouse conditions and watered daily. When the potted plants reached approximately 20 cm in height, 1.1-m bamboo sticks were inserted into the soil of the pots as climbing media for the plants. After growing to 40–50 cm height, the potted plants were evenly divided into two groups, and each group contained 15 plants per species. One group was allowed to continue to grow under normal conditions, serving as the control. The other group was subjected to artificial defoliation treatment for 30 days. During the treatment, all leaves were removed, including the newly grown young leaves. Stem lengths of both invasive and non-invasive species were measured at 10 days intervals. Gas exchange, chlorophyll fluorescence was determined on stems of the invasive and non-invasive species on 20 days after removal of leaves, and anatomic structures of the stems was observed by optical microscope. The survival rates of each species were investigated on 30 days after removal of leaves. The average survival rates were calculated from three biological replicates.

To examine how stem photosynthesis changed in the invasive species, another batch of potted plants of the invasive species was prepared and treated as mentioned above. The changes in photosynthetic pigments (chlorophyll) and non-photosynthetic pigments (anthocyanins), stomatal behavior, gas exchange and chlorophyll fluorescence, photosynthesis-related proteins (D1 and Rubisco) and chloroplast ultrastructure of the stems of the defoliation group were compared with those of the stems and leaves of the non-defoliation group.

### Isolated Stem Culture

The uniform stems of the invasive and non-invasive species were detached from potted plants that had been cultivated for two months. The stems were washed with tap water and cut into segments with a length of approximately 10 cm, each contained at least one node. Five to six segments were placed into a 9 cm petri dish with two pieces of filter paper and 8 mL of distilled water, and then transferred into an RXZ growth chamber (Jiangnan Equipment Factory, Ningbo, China). Cultures were maintained at 28°C/25°C (day/night) under a light/dark photoperiod of 14:10 h, irradiation of ∼40 μmol photon m^–2^ s^–1^ and 70% relative humidity. The isolated stem culture experiments were performed in 4 biological replicates. After 20 days of culture, the survival rate (%) and maximum root length (cm) of stem segments in each petri dish were investigated. Electron transport rate (ETR) of each segment at a photosynthetically active radiation (PAR) of 800 μmol m^–2^ s^–1^ was measured using a chlorophyll fluorescence imaging (CFI) system (Technologica, United States).

### Determination of Gas Exchange

An LI-6800 portable infrared gas analyzer (LI-COR, Inc., United States) was used to measure gas exchange parameters in the stems and leaves. Measurements of the net photosynthetic rate (*P*_*n*_), stomatal conductance and the dark respiration rate (*R*_*d*_) were conducted between 09:00 and 12:00 h on clear days at 800 and 0 μmol photon m^–2^ s^–1^, respectively. PAR was emitted from a red:blue (9:1) LED light source integrated into the LI-6800 measurement chamber (1 × 3 cm). The CO_2_ concentrations flowing into the leaf chamber were controlled at 400 μmol mol^–1^, the temperature of the plant organ in the leaf chamber was maintained at 27°C, and the relative humidity was 65%. Stems of the third internode were selected to analyze the gas exchange. Before the gas exchange measurements were performed, the diameter of the stems was measured using a 150-mm Vernier caliper (Tricle brand, Shanghai, China) and used to compute the projected area. The total stem surface area was calculated by multiplying the projected area by π (3.14). Because the photosynthesis abilities of stems are low, the measurement precision of the device on the stems was much lower than that for the leaves (Supplementary Figure S1). To ensure the accuracy of the stem photosynthesis, the data was acquired at 3–5 s intervals for 2 min using automatic data logging system, and then calculated the mean value of the set of data. Since PAR was applied to only one side of the stems in the measurement chamber, the photosynthesis rate of stems was expressed on a half-surface area basis. However, because stomata are randomly distributed on the stem surface, the stomatal conductance of stems was expressed on a total surface area basis. For the leaves, the photosynthesis rate was expressed on a regular leaf area basis. Because the leaves of the invasive species have stomata on both the abaxial and adaxial sides, to facilitate comparisons with stems, the stomatal conductance of the leaves was expressed on a double-leaf area basis. The gross photosynthesis rate (*P*_*g*_) was subsequently calculated as *P*_*n*_ + *R*_*d*_.

### Determination of Chlorophyll a Fluorescence

Chlorophyll a fluorescence in the stems and leaves was measured by using a chlorophyll fluorescence imaging (CFI) system (Technologica, United States). Plants were adapted to the dark for 40–50 min, after which the stems of the third internode were detached and placed in the measurement chamber of the CFI system. First, the minimum fluorescence (*F*_*o*_) and the maximum fluorescence (*F*_*m*_) stimulated by a 6,000 μmol m^–2^ s^–1^ saturating pulse were measured, and the ratio of variable fluorescence to maximum fluorescence (*F*_*v*_/*F*_*m*_, calculated as 1–*F*_*o*_/*F*_*m*_), which represents the maximum efficiency of photosystem II (Oxborough and Baker, 1997), was calculated. Light curves of chlorophyll a fluorescence were measured under the following PAR amounts: 50, 100, 200, 400, 600, 800, 1,000, and 1,200 μmol m^–2^ s^–1^. The stems were adapted for 90 s at each irradiance, after which the steady fluorescence (*F*) and maximum fluorescence (*F*_*m*_′) in the light-adapted state were measured. The effective quantum yield of PSII (Φ_*PSII*_) was calculated as Φ_*PSII*_ = Δ*F*/*F*_*m*_′ = (*F*_*m*_′ − *F*)/*F*_*m*_′ (Genty et al., 1989). In addition, the ETR was calculated as Φ_*PSII*_ × PAR × 0.85 × 0.5, where the coefficient 0.85 is the PAR absorptivity by plant photosynthetic tissues and where the coefficient 0.5 indicates that the absorbed PAR was equally allocated between PSI and PSII (Krall and Edwards, 1992).

### Determination of Photosynthetic and Non-photosynthetic Pigment Contents

The procedure for the determination of chlorophyll (Chl) was as follows. Three 2-cm-long stem segments or 10-mm leaf disks were homogenized in 4 mL of 80% acetone using a mortar and pestle and then centrifuged at 8,000 g and 4°C for 10 min. The contents of Chl *a*, Chl *b* and the total Chl in the supernatant were determined and calculated according to the methods of Wellburn (1994) and expressed as micrograms per unit area (cm^2^). Since Chl was mainly distributed on the surface layer of stems, the surface area was used in the calculation of Chl contents.

Anthocyanins in the stems and leaves were extracted with methanol-HCl (99:1, v/v) (Craker et al., 1971). Three 1-cm-long stem segments or 1-cm leaf disks were submerged in 4 mL of methanol-HCl overnight in the dark. The Chl in the extract was removed by the addition of 4 mL of chloroform and 1.5 mL of deionized water. The absorbance of the anthocyanin extract at 530 nm was measured. Anthocyanin concentrations were calculated by using a standard curve constructed by cyanidin-3-*O*-glucoside (5–200 μM) and ultimately expressed as micrograms per unit surface area (cm^2^).

### Analysis of D1 and Rubisco Protein Contents

To extract D1 protein, fresh stems or leaves (0.3 g) were homogenized in 2 mL of radioimmunoprecipitation assay (RIPA) buffer containing 1 mM PMSF and the proper amount of protease inhibitor in a precooled mortar, kept in an ice bath for 2 h, and then centrifuged at 12,000 × *g* for 10 min at 4°C. The supernatant containing D1 protein was temporarily stored at 4°C. To extract Rubisco, fresh stems or leaves (0.1 g) were homogenized in 1.5 ml of 60 mM Tris-HCl (pH 7.8) buffer consisting of 5% PVP (w/v), 0.1% NaCl (w/v) and 2% glycerol (v/v) in an ice bath and subsequently centrifuged at 12,000 × *g* for 10 min at 4°C (Zhang et al., 2016). Rubisco proteins were distributed in the supernatant. The proper quantity (0.1 mL) of the extract of D1 protein or Rubisco and an equal volume of protein loading buffer were mixed together, incubated at 100°C for 5 min and then stored at 4°C for further analysis. An aliquot of each sample (20 μl) was loaded into one well of an SDS-PAGE gel prepared in advance (Zhang et al., 2016), and the proteins were separated by SDS-PAGE with a Mini-PROTEAN 3 system (Bio-Rad, United States). The separated proteins in the gels were blotted onto PVDF membranes, and the membranes were stained with Ponceau S to confirm equal protein transfer. The membranes were then washed three times (5 min each) in TBST (50 mM Tris [pH 7.5], 150 mM NaCl, 0.1% Tween 20) and subsequently blocked with 5% (w/v) non-fat powdered milk in TBST for 1.5 h. The membranes containing D1 protein and the large subunit of Rubisco were incubated together with PsbA global antibodies (1:10,000 dilution) (Agrisera, Sweden) and anti-Rubisco antibodies (1:1,000 dilution) (Bioss, Beijing, China) overnight at 4°C, respectively. The blots were washed with TBST three times and then incubated with goat anti-rabbit HRP-conjugated secondary antibodies (1:3,000 dilution) at room temperature for 50 min. The blots were subsequently washed three times in TBST, and protein visualization was performed using a Tanon 5200 enhanced chemiluminescence (ECL) detection system (Tanon, Shanghai, China).

### Microscopy-Based Inspection of the Stems

The surface and cross-sectional characteristics of the stems of both the invasive and non-invasive species were observed and imaged via a super resolution system (Liyang Precision Machinery, Chengdu, China). To obtain a high resolution image of the surface structure of stems, 20 multi-focus images with shallow depth of field were taken for each stem, and fused into a higher resolution image using the Zerene Stacker software (Zerene Systems LLC, Richland, WA, United States).

### Measurement of Stomatal Aperture

Main stems of the invasive species were detached between 09:00 and 10:00 h and brought into the lab with the cut end submerged in distilled water. Epidermal layer of the stems of the third internode and mature leaves (including adaxial side and abaxial side) of the invasive species was torn off with fine-tipped tweezers, made wet mount microscope slides and viewed using a Leica DM6000 microscope (Leica, Wetzlar, Germany). Digital micrographs of the epidermal surfaces (20× and 40× magnification) were captured and used to quantify stomatal density and stomatal aperture. The image dimensions were calibrated using the objective micrometer. Stomatal densities were analyzed using the images captured through a the 20× objective and expressed on an area basis (stomata mm^–2^). The stomatal density of each sample was calculated as the average of four images that were captured from different fields of view. The stomatal lengths and widths were determined using the images captured through the 40× objective, and for each treatment, at least 20 stomata were measured.

### Observations of Chloroplast Ultrastructure

Fresh stems of the third internode and mature leaves were cut into approximately 1 mm × 1 mm pieces, vacuum infiltrated and fixed in 2.5% glutaraldehyde and 2% paraformaldehyde in 0.1 M phosphate buffer (pH 7.0). The samples were then dehydrated in a graded series of ethanol and embedded in Epon 812 epoxy resin. The embedded samples were sliced into ultrathin sections approximately 70 nm in thickness using an ultramicrotome (Leica UC7, Leica), double-stained with uranium lead (a 2% uranyl acetate and lead citrate saturated aqueous solution) and observed under a transmission electron microscope (HT7700, Hitachi Japan). The length and width of the chloroplasts were determined from the images captured at 1,200× magnification, and 10 chloroplasts were measured for each treatment.

### Statistical Analysis of the Data

Statistical significance was determined by one-way ANOVA followed by Duncan *post hoc* tests using IBM SPSS Statistics 19.0 (IBM, Armonk, NY, United States). The means were considered to be significantly different at the level *P* < 0.05 according to the *post hoc* test. Before performing ANOVA, the data were checked for normality and homogeneity of variance; if the assumptions were not met, the data were log-transformed and retested. Student’s test was used to analyze the significance of the changes in stem photosynthesis in the invasive plants after defoliation. The data are presented as the means and standard errors.

## Results

### Differences in Plasticity of Stem Photosynthesis and Relevant Traits in Response to Defoliation Between *M. micrantha* and Non-invasive Species

**FIGURE 1:**
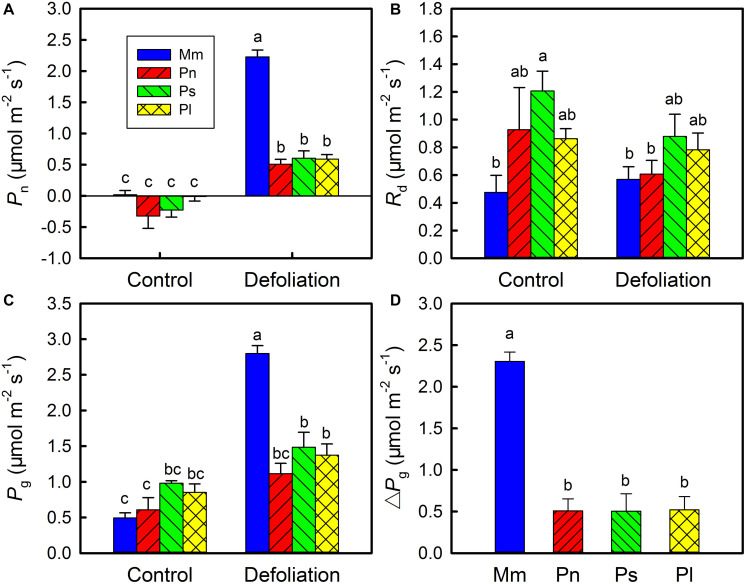
Changes in net photosynthetic rate (*P*_*n*_) **(A)**, day respiration rate (*R*_*d*_) **(B)**, gross photosynthetic rate (*R*_*g*_) **(C)** in stems of *Mikania micrantha* (Mm), *Pharbitis nil* (Pn), *Paederia scandens* (Ps), and *Pueraria lobata* (Pl) after removal of leaves for 20 days. The increasing extent of gross photosynthetic rate relative to the control **(D)** in stems of the four species after removal of leaves for 20 days. The error bars represent the standard errors (SE) of four to five biological replicates. The bars with different letters (a, b, c) indicate significant differences between the means (Duncan’s multiple range test, *P* < 0.05).

To explore the plasticity of stem photosynthesis in *M. micrantha*, the leaves of *M. micrantha* and three non-invasive species (i.e., *Pharbitis nil*, *Paederia scandens*, and *Pueraria lobata*) were removed. In the control group, no significant differences in net photosynthetic rate (*P*_*n*_) and gross photosynthetic rate (*P*_*g*_) were found between the invasive species and the three non-invasive species, although the dark respiration rate (*R*_*d*_) of the invasive species was lower than those of the three non-invasive species (Figures 1A–C). After defoliation, both *P*_*n*_ and *P*_*g*_ of the invasive species and non-invasive species increased significantly, but the *R*_*d*_ remained relatively constant. The increases in *P*_*n*_ and *P*_*g*_ in the invasive species were significantly greater than those in the non-invasive species. On day 20 of defoliation, the increase in *P*_*g*_ (Δ*P*_*g*_) in the invasive species reached 2.4 μmol m^–2^ s^–1^, while that of the three non-invasive species reached only 0.5 μmol m^–2^ s^–1^ (Figure 1D), indicating that stem photosynthesis in the invasive species is more responsive than that in the non-invasive species.

**FIGURE 2:**
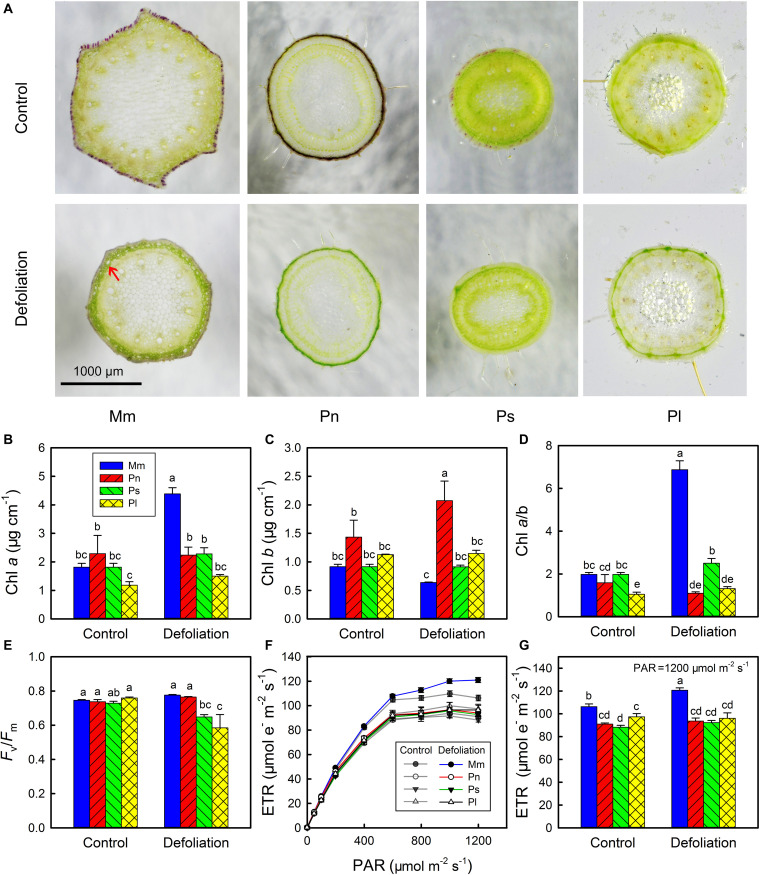
Changes in transverse section of stems of *Mikania micrantha* (Mm), *Pharbitis nil* (Pn), *Paederia scandens* (Ps), and *Pueraria lobata* (Pl) after removal of leaves for 20 days **(A)**. Changes in chlorophyll (Chl) *a* content **(B)**, Chl *b* content **(C)**, Chl *a*/*b* ratio **(D)**, maximal quantum yield (*F*_*v*_/*F*_*m*_) **(E)**, light response curve of electron transport rate (ETR) **(F)**, and ETR at photosynthetically active radiation (PAR) of 1,200 μmol mm^–2^ s^–1^ **(G)** in stems of the four species after removal of leaves for 20 days. The error bars represent the standard errors (SE) of four to five biological replicates. The bars with different letters (a, b, c, d, e) indicate significant differences between the means (Duncan’s multiple range test, *P* < 0.05).

According to the cross-sections, the stem epidermis of *M. micrantha*, *P. nil*, and *P. scandens* evidently contained anthocyanin pigments, and these pigments disappeared with the removal of leaves (Figure 2A). Though *P. lobata* did not contain anthocyanins, it had more epidermal pubescence than the other species did, and the epidermal pubescence was not affected by defoliation. In addition to the disappearance of anthocyanin pigments, the 3–4 layers of cells below the stem epidermis of *M. micrantha* became more apparent (indicated by the red arrow). These layers of cells became larger and contained more chlorophyll, which formed a conspicuous green ring zone. However, no similar structural change was found in the non-invasive species.

The results of the chlorophyll and chlorophyll a fluorescence measurements showed that there were no significant differences in Chl *a*, Chl *b*, the Chl *a*/*b* ratio and *F*_*v*_/*F*_*m*_ between the invasive species and the non-invasive species (Figures 2B–E). However, the invasive species had a greater ETR than the three non-invasive species did, especially when the PAR was greater than 400 μmol m^–2^ s^–1^ (Figures 2F,G). With the removal of leaves, the Chl *a* content sharply increased in the stems of *M. micrantha*, but the Chl *b* content remained at a relatively constant state. By contrast, both Chl *a* and Chl *b* contents showed little change in the non-invasive species, except that there was a slight increase in Chl *b* in *P. nil*. The marked increase in Chl *a* in the stems of *M. micrantha* resulted in their Chl *a*/*b* ratio being higher than that of the non-invasive species. Accompanied by the increases in the Chl *a* content and the Chl *a*/*b* ratio, the ETR also increased in the invasive species. Thus, the morphology, photosynthetic pigments and photochemistry of the stems in the invasive species were more dynamic than were those of the non-invasive species.

### Differences in Effects of Defoliation on the Elongation and Survival of Stems Between the Invasive and Non-invasive Species

**FIGURE 3:**
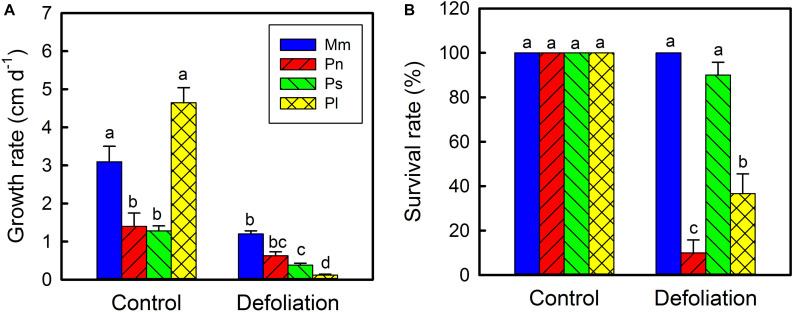
Changes in stem growth rate of *Mikania micrantha* (Mm), *Pharbitis nil* (Pn), *Paederia scandens* (Ps), and *Pueraria lobata* (Pl) after removal of leaves **(A)**. Survival rate of the four species after removal of leaves for 30 days **(B)**. The error bar represents the standard error (SE) of three to five biological replicates. The bars with different letters (a, b, c, d) indicate significant differences between the means (Duncan’s multiple range test, *P* < 0.05).

Under controlled environmental conditions, the average elongation rate of stems of *M. micrantha* was significantly greater than that of *P. nil* and *P. scandens* but was not significantly different from that of *P. lobata* (Figure 3A). With the removal of leaves, the stem elongation rates of all the species decreased by varying degrees, and those of *M. micrantha* were 0.91–9.07 times higher than those of the three non-invasive species. On day 30 of defoliation, the survival rate of the *M. micrantha* plants reached 100%, while those of non-invasive species ranged from 10 to 90% (Figure 3B).

### Differences in Survival Rate and Growth Characteristics of the Stems Between the Invasive and Non-invasive Plants in Isolated Culture

**FIGURE 4:**
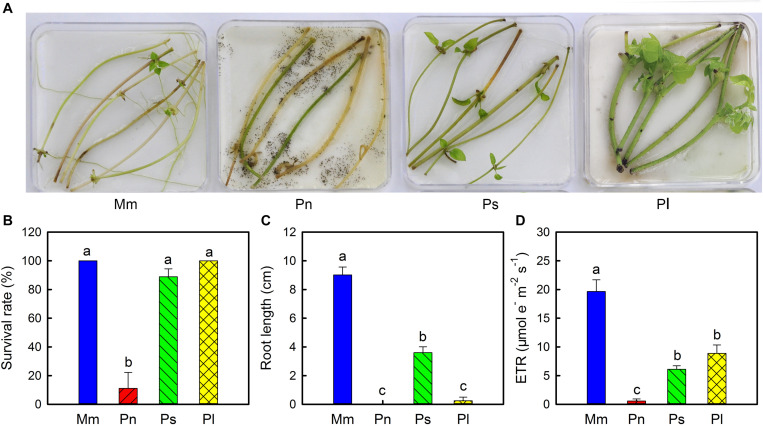
Phenotype **(A)**, survival rate **(B)**, root length **(C),** and electron transport rate (ETR) **(D)** of stem segments of *Mikania micrantha* (Mm), *Pharbitis nil* (Pn), *Paederia scandens* (Ps), and *Pueraria lobata* (Pl) after cultured 20 days in the petri dishes. The error bar represents the standard error (SE) of three to six biological replicates. The bars with different letters (a, b, c) indicate significant differences between the means (Duncan’s multiple range test, *P* < 0.05).

To further confirm that stem viability of the invasive species was different from that of the non-invasive species, their detached stem segments were cultured in petri dishes in a growth chamber (Figure 4). After 20 days of culturing, the survival rates of *M. micrantha*, *P. lobata*, *P. scandens*, and *P. nil* were 100, 100, 90, and 11%, respectively (Figure 4B). In isolated culture conditions, *M. micrantha* preferentially developed roots compared with shoots, but the opposite was true for *P. lobata* and *P. scandens*. As a result, the length of *M. micrantha* roots was much greater than the length of the roots of the three non-invasive species (Figure 4C). Compared with the non-invasive species, *M. micrantha* had a higher ETR (Figure 4D), which was in agreement with the results observed in the defoliation treatment.

### Mechanism Governing the Upregulation of Photosynthesis in Stems of Invasive Species After Defoliation

**FIGURE 5:**
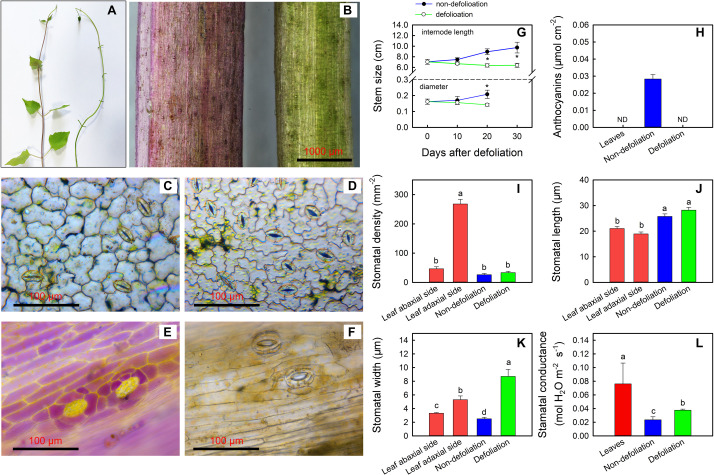
Changes in stem phenotype of *Mikania micrantha* after removal of leaves for 20 days **(A,B)**. Stomata on the abaxial side **(C)** and adaxial side **(D)** of leaves of the non-defoliation group. Stomata on stems of the non-defoliation **(E)** and defoliation groups **(F)**. Changes in stem internode length and stem diameter in the non-defoliation and defoliation group **(G)**. Comparisons of anthocyanin content **(H)**, stomatal density **(I)**, stomatal length **(J)**, stomatal width **(K)**, and stomatal conductance **(L)** in stems of *Mikania micrantha* after removal of leaves for 20 days with those in leaves and stems of the non-defoliation group. The error bar represents the standard error (SE) of four to five biological replicates. The bars with asterisks indicate significant differences between the means (*t*-test, *P < 0.05). The bars with different letters (a, b, c) indicate significant differences between the means (Duncan’s multiple range test, *P* < 0.05).

As described above, the largest upregulation of stem photosynthesis was found in *M. micrantha* among the four species. To determine the regulatory mechanism governing stem photosynthesis of the invasive species, morphological and physiological changes were further investigated (Figures 5A–L). During the defoliation treatment, the average internode lengths of the stems became shorter, and the average diameter of the stems noticeably decreased (Figure 5G). On day 20 of defoliation, anthocyanins were no longer detected in the stems (Figure 5H). We noted that leaves of non-defoliation group had stomata on both the adaxial and abaxial surfaces, and the stomatal density (270 stomata mm^–2^) on the abaxial surface was five times that on the adaxial surface. The density of stomata on the surface of stems was comparable to that on the adaxial surface of the leaves (Figure 5I). However, the stomata on the stems were evidently larger than those on the leaves. The stomatal density of the stems was not influenced by the defoliation treatment, whereas the stomatal aperture and stomatal conductance increased significantly (Figures 5I–L). The width of the stomata on the stems of defoliated plants was even larger than that on either the adaxial surface or abaxial surface of the leaves of the control plants. However, the stomatal conductance of stems of the defoliated plants was still significantly lower than that of the leaves because the leaves had a greater average stomatal density.

**FIGURE 6:**
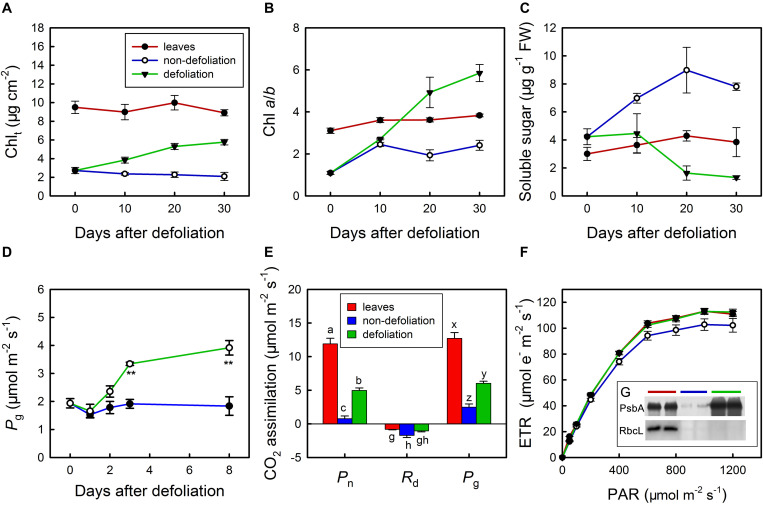
Changes in total chlorophyll content (Chl_*t*_) **(A)**, chlorophyll *a*/*b* ratio **(B)**, soluble sugar content **(C)** in stems of *Mikania micrantha* after removal of leaves compared with those in stems and leaves of the non-defoliation group. Changes in gross photosynthetic rate (*P*_*g*_) in stems of *Mikania micrantha* after removal of leaves **(D)**. Comparisons of net photosynthetic rate (*P*_*n*_), day respiration rate (*R*_*d*_), gross photosynthetic rate (*P*_*g*_) **(E)**, light response curve of electron transport rate (ETR) **(F)**, relative quantification of D1 protein and Rubsico **(G)** in stems of *Mikania micrantha* after removal of leaves for 20 days with those in leaves and stems of the non-defoliation group. The error bars represent the standard errors (SE) of four to five biological replicates. The bars with asterisks indicate significant differences between the means (*t*-test, **P < 0.01). The bars with different letters (a, b, c; g, h; x, y, z) indicate significant differences between the means (Duncan’s multiple range test, *P* < 0.05). FW, flesh weight; PAR, photosynthetically active radiation; PsbA, D1 protein of PSII; RbcL, the large subunit of ribulose-1,5-bisphosphate carboxylase/oxygenase (Rubisco).

Under normal conditions, the total chlorophyll (Chl_*t*_) content per unit area and the Chl *a*/*b* ratio in the stems were equal to a quarter and a half of those in the leaves, respectively (Figures 6A,B). In addition, the soluble sugar content in the stems of non-defoliation group was also significantly lower than that in the leaves (Figure 6C). With the removal of leaves, both the total Chl content the Chl *a*/*b* ratio gradually increased in the stems; however, the soluble sugar level showed a decreasing trend. On day 10 of defoliation, the differences in total Chl in the stems between the defoliation group and non-defoliation group had reached a significant level. Though the total Chl content continued to increase in the stems of defoliated plants during the following 20 days, it was consistently lower than that in the leaves of non-defoliation group. By contrast, the Chl *a*/*b* ratio of the stems of the defoliated plants was found to exceed that of leaves (non-defoliation group) on day 20 of defoliation. In fact, photosynthesis of the stems responded to defoliation much faster than the chlorophyll and soluble sugar contents did. On day 3 of defoliation, the gross photosynthesis rate of the stems was greater than that of non-defoliation group, and continued to increase during the next several days (Figures 6D,E). The ETR also significantly increased in the stems 20 days after defoliation, reaching a level that was equal to that of leaves non-defoliation group (Figure 6F). In addition, the D1 protein (PsbA) of PSII was dramatically upregulated in the stems, and its concentration even exceeded that in the leaves, but the RbcL protein content appeared to decrease (Figure 6G; inserted in Figure 6F). Therefore, the increase in stem photosynthesis of *M. micrantha* was due to multiple factors that improved the gas exchange efficiency of the stems and the ability of chloroplasts to absorb light energy.

**FIGURE 7:**
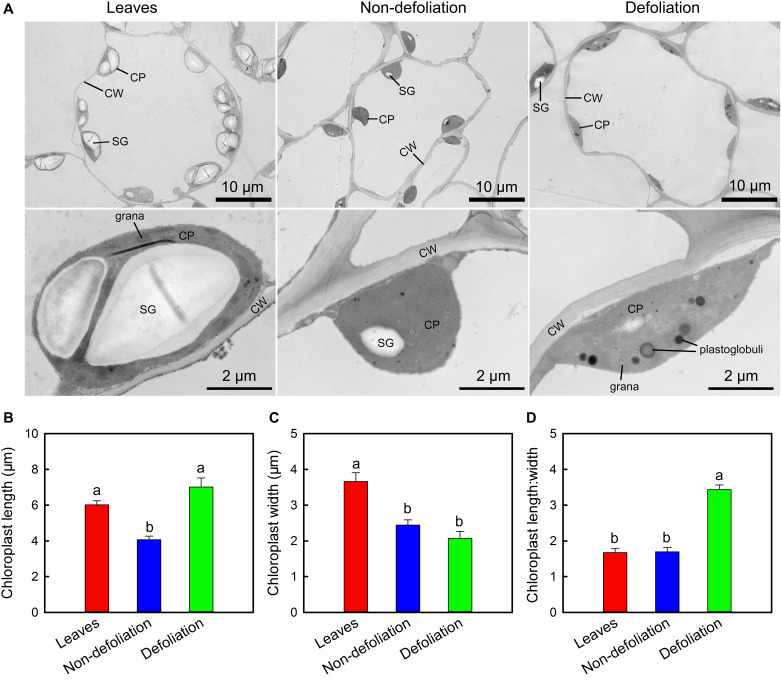
Comparisons of subcellular structure of stems of *Mikania micrantha* after removal of leaves for 20 days with those of leaves and stems of the non-defoliation group **(A)**. Comparisons of chloroplast length **(B)**, width **(C)**, and lengh:width ratio **(D)** in stems of *Mikania micrantha* after removal of leaves for 20 days with those of leaves and stems of the non-defoliation group. The error bars represent the standard errors (SE) of six biological replicates. The bars with different letters (a, b) indicate significant differences between the means (Duncan’s multiple range test, *P* < 0.05). CP, chloroplast; CW, cell wall; SG, starch grain.

Ultrastructural observations revealed that the chloroplasts in the stems and leaves of intact *M. micrantha* plants were elliptical shaped and contained 1–2 starch grains (Figure 7A). The starch grains occupied more than 50% of the interior space of the leaf chloroplasts, but those that accumulated in the stem chloroplasts were much smaller. Defoliation significantly affected the structure of the stem chloroplasts. The longitudinal section of the chloroplasts changed from being elliptical shaped to spindle shaped. As the length increased by 72.5% and the width decreased by 15.2%, the length:width ratio of the chloroplasts in the defoliated plants was approximately two-fold that of the control plants (Figures 7B–D). Such changes might increase the light-reception area of the chloroplasts. In addition to the change in shape, the number and size of plastoglobuli in the stem chloroplasts of defoliated plants were also larger than those in the intact plants, the diameter of the former could reach up to 0.44 μm.

## Discussion

### Effects of Stem Physiological Plasticity on the Growth of *Mikania micrantha*

Understanding the ecophysiological mechanisms underlying species invasion is required to perform effective management. Morphological and physiological plasticity associated with the broad expansion of various invasive species has been verified by a large number of empirical studies (Richards et al., 2006; Riis et al., 2010; Geng et al., 2016). For invasive vines, morphological plasticity of internode length and internode number of the stems may allow these plants to actively occupy favorable microhabitats, ultimately affecting plant fitness (Schweitzer and Larson, 1999). However, little is known about the physiological plasticity of stems in invasive vine species.

In addition to leaves, non-foliar organs such as stems, bark, and fruits of many higher plants can also carry out photosynthesis (Xu et al., 1997; Tarvainen et al., 2018). Although the photosynthesis ability of non-foliar organs is often significantly less than that of leaves, it still plays an important physiological role in the growth, development and stress resistance of plants. For example, stem photosynthesis can increase stem growth (Cernusak and Hutley, 2011; Steppe et al., 2015), improve the carbon economy of whole plants (Nilsen, 1995) and improve drought resistance (Vandegehuchte et al., 2015). In this study, we used a gas exchange analysis technique to confirm that the stems of *M. micrantha* and non-invasive species were able to perform photosynthesis because dramatically more CO_2_ was released from the stems in the dark than in the light (Supplementary Figure S1). Our results also revealed that the stem cortex of the vine species was permeable to CO_2_. This is unlike that in woody species, as the cambium in the stems of these species is gas impermeable and blocks radial CO_2_ diffusion (Steppe et al., 2007). As a result, stem photosynthesis in woody species can be used only to refix respired CO_2_, but in vine species, stem photosynthesis can fix CO_2_ from both cellular respiration and ambient air. In evolutionary terms, stem photosynthesis in vine species is much more similar to leaf photosynthesis than that in woody species.

Stem photosynthetic ability and its impact on fitness varied from species to species (Berveiller et al., 2007). In the current study, compared with the three non-invasive species, *M. micrantha* manifested a greater plasticity of stem photosynthesis, as reflected by changes in the net photosynthetic rate, total photosynthetic rate, Chl *a* content, and ETR during defoliation (Figures 1, 2). Related to these physiological characteristics, compared with the three non-invasive species, *M. micrantha* demonstrated a higher stem elongation rate during the leaf defoliation treatment (Figure 3). Moreover, the survival rate of the defoliated *M. micrantha* plants reached 100%, while the rates of the non-invasive species ranged from 10 to 90%, suggesting that physiological plasticity of the stems plays an important role in maintaining the survival and growth of *M. micrantha* under defoliation. Similarly, under the conditions of isolated culture, *M. micrantha* showed higher stem photosynthesis than did the non-invasive species, as indicated by the ETR (Figure 4). As a result, the survival rate of *M. micrantha* stem segments was 100%, which was higher than that of two of the three non-invasive species. We noted that the stem segment-regeneration strategy adopted by *M. micrantha* was different from that adopted by the non-invasive species (Figure 4). *M. micrantha* preferentially rooted first, whereas the non-invasive species *P. lobata* and *P. scandens* tended to grow leaves first; moreover, *P. nil* segments could hardly regenerate. This may also be associated with stem photosynthesis performance. Compared with those of the non-invasive species, the stem segments of *M. micrantha*, which a higher photosynthesis rate, produced more carbohydrates, setting the stage for the stem cuttings of *M. micrantha* to root first. The growth of roots allows the regenerated plants to rapidly uptake water and nutrients, thereby increasing the probability of survival. In contrast, the photosynthesis ability of the stems of the non-invasive species was lower, so only prioritizing the growth of new leaves as photosynthetic organs can guarantee supplies of carbohydrates for further growth of the regenerated plants.

### Modified Mechanism of Stem Photosynthesis in *M. micrantha*

Plants obtain carbohydrates from photosynthesis and consume them via respiratory processes for maintaining metabolism (McDowell and Sevanto, 2010). The removal of leaves stops the primary carbohydrate source from plants, causing them to experience carbon stress. Defoliation-induced carbon stress can reduce non-structural carbohydrate reserves and increase both vulnerability to insect infestation and hydraulic performance (Anderegg and Callaway, 2012). However, in some cases, a reduction in leaf photosynthesis can be compensated for by the mobilization of stored carbohydrates, reallocation of carbon or stem photosynthesis (Eyles et al., 2009). Compared with leaf photosynthesis, stem photosynthesis is less vulnerable to environmental stresses such as seasonal changes and reduced water availability; therefore, stem photosynthesis is able to compensate for the loss of leaf photosynthesis under stress conditions (Nilsen and Bao, 1990; Nilsen et al., 1993). In *M. micrantha*, the response of soluble sugars to defoliation treatment was slower than the response of stem photosynthesis (Figure 6), suggesting that stem photosynthesis rather than the stored carbohydrates is preferentially used to compensate for the decrease in leaf photosynthesis.

The carbon starvation-induced optimization of stem photosynthetic ability in *M. micrantha* is an integration of a series of interactive factors. In terms of the phenotype, removal of the leaves decreased the internode length, diameter and anthocyanin pigmentation of the stems (Figure 5). Although internode length is unrelated to stem photosynthesis ability, a shorter internode length could provide more opportunities for sprouting new leaves along the stems. By contrast, thinner stems have a larger surface area-to-volume ratio that favors gas exchange between the stems and external environment. However, as the low projected area of thin stems may affect their light capture ability. Interestingly, this deleterious effect could be offset by reducing the amount of anthocyanin pigments, which act as light barriers, in the stem epidermis.

Anthocyanin pigments can act as light attenuators or as antioxidants to protect the photosynthetic apparatus in plant vegetative tissues (Neill and Gould, 2003). Since anthocyanins were distributed in the epidermis of the stems of the plants in the present study, they were more likely to play a light-barrier role rather than an antioxidant role. In fact, anthocyanins accumulate in young stems but diminish with maturity. This pigmentation pattern has a lot in common with that in leaves (Hughes et al., 2007; Zhu et al., 2018), suggesting that different kinds of tissues can utilize the same approach to protect immature photosynthetic apparatuses. With regard to the non-invasive species, *P. nil* and *P. scandens* had the same pigmentation pattern in their stems as *M. micrantha* did (Figure 2 and Supplementary Figure S2). The stems of *P. lobata* did not accumulate anthocyanins, but they had more pubescence than the stems of the other species did. Pubescence can play a similar role as anthocyanins with regard to photoprotection (Liakopoulos et al., 2006). Defoliation induced an improvement in the stem photosynthesis ability, allowing the chloroplasts to withstand stronger irradiance. In this case, anthocyanins become an unnecessary tool to protect the stem photosynthetic apparatus. After removal of leaves, the reduction in anthocyanins in the stems was observed to be associated with decreased soluble sugar contents, indicating that sugars are involved in the regulation of anthocyanins. This is consistent with the findings of a recent study showing the regulatory mechanism of anthocyanins in kiwifruit (Nardozza et al., 2020).

In addition to the removal of light-barrier pigments, carbon starvation also induced stomatal opening and thereby removed the obstacle that limited gas exchange between the stems and the atmosphere. Stomatal opening was confirmed by microscopy-based observations and measurements of stomatal conductance based on H_2_O exchange (Figure 5). However, because the stomatal densities were different, stomatal conductance in the stems without leaves was observed still to be lower than that in the leaves of non-defoliation plants.

In addition to overcoming the barriers to light and CO_2_, *M. micrantha* was also able to optimize its cellular and subcellular structure to increase photosynthesis efficiency. There were 3–4 layers of cells below the epidermis that became larger and accumulated increased amounts of chlorophyll, indicating that photosynthesis was mainly performed in this zone of the stems (Figure 2). Ultrastructural observations showed that chloroplasts in this zone of the intact plants were elliptica shaped; however, after the removal of their leaves, they became spindle shaped, and their length dramatically increased (Figure 7). Such changes might increase the light-receiving area of the chloroplasts. The modification of the Chl *a*/*b* ratio and plastoglobule size and the enhanced turnover of D1 protein supported that chloroplasts were exposed to high light in the stems of the defoliated plants.

Plastoglobuli are lipoprotein particles present in both non-photosynthetic and photosynthetic plastids in plants (van Wijk and Kessler, 2017). In chloroplasts, they play functional roles in chloroplast biogenesis, redox and photosynthetic regulation and senescence by exchanging metabolites with the thylakoid membrane (Vom Dorp et al., 2015). They can serve as extrathylakoid storage sites for excess isoprenoid lipids, such as α-tocopherol (vitamin E), plastoquinone-9, and traces of xanthophylls (Lichtenthaler, 2007). Under high-light conditions, as the essential components of thylakoids, α-tocopherol and plastoquinone-9 often accumulate in excess amounts in chloroplasts and are stored in plastoglobuli. The degradation of epidermal anthocyanins and changes in chloroplast shape caused the chloroplasts in the stem tissues to be exposed to high light. This could explain why, compared with those of intact plants, the chloroplasts in the stems of the defoliated plants had a higher number and a larger size of plastoglobuli (Figure 7).

During defoliation, the Chl *a* content increased rapidly in the stems of *M. micrantha*, whereas the Chl *b* content was maintained at a similar level (Figure 2). As a consequence, the Chl *a*/*b* ratio dramatically increased, which is in agreement with the findings in leaves exposed to high irradiance (Kitajima and Hogan, 2003; Sarijeva et al., 2007). The differences in Chl *a*/*b* ratios between sun and shade leaves are due to the high-irradiance-adaptation response of the photosynthetic machinery of sun leaves, which have a much lower quantity of light-harvesting Chl *a*/*b* proteins (LHCII) and a greater number of PSII cores than shade leaves do (Lichtenthaler et al., 1982). Since the Chl *a*/*b* ratio was found to be positively correlated with the ratio of PSII cores to LHCII (Terashima and Hikosaka, 1995), this ratio is used as an indicator of N partitioning within a leaf (Kitajima and Hogan, 2003). It is likely that the stems experienced N limitation during defoliation, as the removal of leaves caused energy starvation in the roots, which could in turn reduce the uptake of N. If so, the adjustment of the stem Chl *a*/*b* ratio during the removal of leaves was a result of irradiance and N availability acting together. In fact, differences in N partitioning in stem chloroplasts between defoliated plants and intact plants could be confirmed by the fact that the stems of the defoliated plants had more D1 protein and a lower amount of soluble protein than the stems of the control plants did (Figure 6). Moreover, the Rubisco content in the defoliated stems also seemed to be slightly reduced compared with that in the stems of intact plants.

The photosynthesis of plants depends on the function of photosystem II (PSII), which is a large multisubunit protein complex integrated within the thylakoid membrane (Andersson and Barber, 1994). The PSII reaction center contains the homologous D1 and D2 proteins, PsbI, PsbW and cytochrome b559. This study showed that the amount of D1 protein dramatically increased in the stems of *M. micrantha* after removal of the leaves (Figure 6). The increase in D1 protein was positively associated with the Chl *a*/*b* ratio, the ETR and Φ_*PS*__*II*_ (Supplementary Figure S3), indicating that more PSII reaction centers were assembled in the stem chloroplasts during defoliation. However, the increase in D1 protein relative to that of the control was evidently greater than the increase in total Chl and the Chl *a*/*b* ratio. In fact, the D1 protein in PSII is prone to irreversible damage caused by reactive oxygen species that are formed in the light, and there is an intricate repair mechanism involving degradation of the damaged D1 reaction center protein and insertion of the newly synthesized copy into the photosystem for maintaining photosynthesis (Lindahl et al., 2000). Generally, the rate of D1 impairment does not exceed the rate of its repair under optimal growth conditions; therefore, no adverse effects on photosynthesis efficiency is manifested. Stress conditions such as high light can disrupt the balance between D1 protein impairment and its repair, resulting in photoinhibition and lowering the quantum yield of photosynthesis and the ETR of PSII (Andersson and Aro, 2006). Thus, D1 protein turnover is crucial for plasticity of the photosynthetic apparatus. In the stems of *M. micrantha*, the high rate of D1 synthesis guaranteed the maintenance of high photosynthesis efficiency in the absence of anthocyanin-mediated photoprotection.

## Conclusion

Our results highlight the importance of physiological plasticity of stems in plant invasiveness. High plasticity of stem photosynthesis improves the survival and fitness of *M. micrantha* compared with non-invasive species under harsh conditions and allows the plants to rapidly recover from defoliation injuries. Many invasive alien plants can be controlled by releasing enemy insects to attack their leaves (Clewley et al., 2012; Havens et al., 2019). This technique may be less effective to manage *M. micrantha*, as this invasive species can enhance its stem photosynthesis to maintain plant survival for a long time. We demonstrated that chloroplast morphology, anthocyanins, stomata, photosynthetic pigments and photosynthesis-related proteins are involved in improving the photosynthesis efficiency of the stems of *M. micrantha* during defoliation. However, the details through which such processes occur are far from clear. The regulatory mechanisms underlying anthocyanin degradation and stomatal behavior in the stems need to be investigated. Moreover, the mechanism through which N partitioning between thylakoid membrane proteins and soluble proteins contributes to improved photosynthesis efficiency should be clarified.

## Data Availability Statement

The original contributions presented in the study are included in the article/Supplementary Material, further inquiries can be directed to the corresponding author/s.

## Author Contributions

JZ and T-JZ conceived the idea, designed the experiment and wrote the manuscript. JZ, B-HL and W-JL carried out the morphological and physiological analyses. Q-LZ and M-LC conducted the western blot analyses. C-LP coordinated the project and revised the manuscript. All authors contributed to the article and approved the submitted version.

## Conflict of Interest

The authors declare that the research was conducted in the absence of any commercial or financial relationships that could be construed as a potential conflict of interest.
