# Supplementary material for: Strong Response of Stem Photosynthesis to Defoliation in Mikania micrantha Highlights the Contribution of Phenotypic Plasticity to Plant Invasiveness
Source: Front Plant Sci. 2021 May 5;12:638796. doi: 10.3389/fpls.2021.638796 (PMC8131553; doi:10.3389/fpls.2021.638796)
Supplement: Supplementary file 1 [file Data_Sheet_1.docx]

**Supporting information**

**Stem photosynthesis in *Mikania micrantha***

A Li6800 portable photosynthesis system was use measured stem photosynthesis in *M. micrantha*. Net photosynthesis of the stems were expressed on a half surface area basis. From **Figure S1**, it can be seen that stem photosynthesis was an eighteenth of leaf photosynthesis. Due to the small effective area and the limited accuracy of the instrument, the stem photosynthesis showed a larger fluctuation than leaf photosynthesis. The maximum fluctuation range stem photosynthesis reached 0.5 μmol m^–2^ s^–1^, which was significant larger than the fluctuation level of the leaves (0.1 μmol m^–2^ s^–1^).

**FIGURE S1**| Changes of net photosynthetic rate in leaves and stems of *Mikania micrantha* when PPFD was switched off (**A**). Net photosynthetic rate and respiration rate in stems of *Mikania micrantha* was plotted together in one figure (**B**). Gas exchange in leaves and stems of *Mikania micrantha* were measured in the intervals of 5 seconds using a LI-6800 Portable Photosynthesis System (Li-Cor, Lincoln, NE, USA).

**Stem surface characteristics of *Mikania micrantha* and the native species**

According to the microscopic image of the stems (**Figure S2**), we can clearly see that the stem surface characteristics of the invasive species *M. micrantha* was different from the native species. The stem surface of *M. micrantha* was smooth and glabrous, whereas those of the three native species had abundant trichomes. *Pueraria lobata* had the longest trichome length, which was followed by that of *Pharbitis nil* and *Paederia scandens*. Under normal growth conditions, *M. micrantha*, *P. nil* and *P. scandens* accumulated anthocyanins leading to a characteristic red of the stems. They turn into green during defoliation treatment. By contrast, *Pueraria lobata* did not accumulate anthocyanins in the stems, and had no visible change during defoliation treatment.


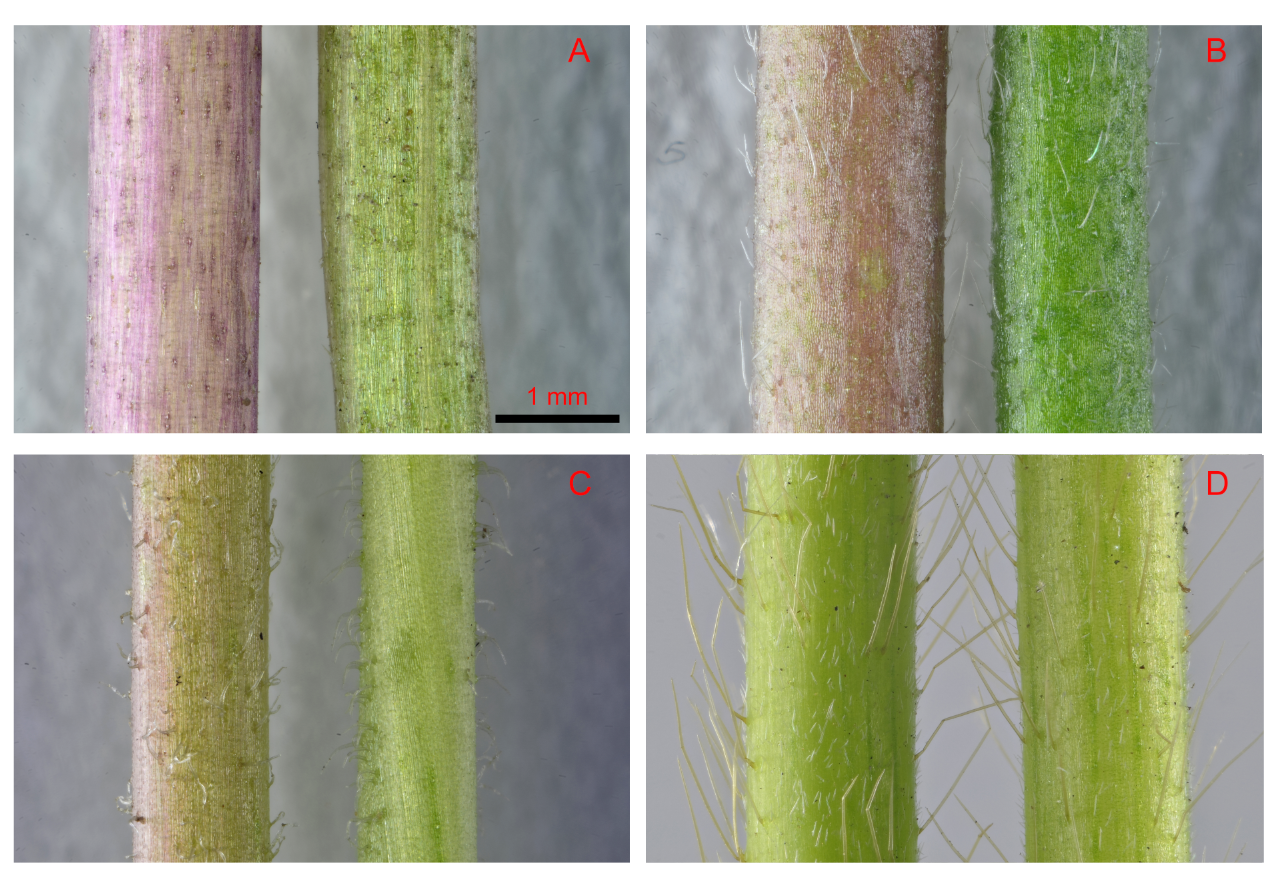


**FIGURE S2**| Stem external features of invasive plant *Mikania micrantha* (**A**) and three native plants, *Pharbitis nil* (**B**), *Paederia scandens* (**C**) and *Pueraria lobata* (**D**) on after removal of leaves for 20 days. In each panel, the stem on the left and right were the non-defoliation group and defoliation group, respectively.

**Changes of Φ_PSII_ and NPQ in the stems of *Mikania micrantha*** **after removal of leaves**

On day 20 after removal of leaves, the effective quantum yield (Φ_PSII_) in stems of *M. micrantha* increased to the levels of its leaves (**Figure S3**). Similarly, non-photochemical quenching (NPQ) was shifted toward the levels of leaves. Therefore, chlorophyll fluorescence characteristics in the stems become more similar as in leaves of non-defoliation plants after removal of leaves.

**FIGURE S3**| Change of effective quantum yield (*Φ*_PSII_) (**A**) and non-photochemical quenching (NPQ) (**B**) in stems of *Mikania micrantha* after removal of leaves for 20 days. Leaves of the non-defoliation group were also used the object to be compared.
